# Analysis of the Maternal and Child Health Care Status in Suizhou City, Hubei Province, China, from 2005 to 2011

**DOI:** 10.1371/journal.pone.0072649

**Published:** 2013-08-15

**Authors:** Cui-Ling Li, Tao Jiang, Xiu-Zhen Hu, Kai Zhao, Qiong Yu, Hui-Ping Zhang

**Affiliations:** 1 Family Planning Research Institute, Tongji Medical College, Huazhong University of Science and Technology, Wuhan, Hubei, China; 2 Suizhou Maternal and Child Health-care Hospital, Suizhou, Hubei, China; University of Alabama at Birmingham, United States of America

## Abstract

**Background:**

Improving the health and well-being of women and children has long been a common goal throughout the world. From 2005 to 2011, Suizhou City had an annual average of 22,405 pregnant and parturient women (1.04% of the population) and 98,811 children under 5 years old (4.57% of the population). Understanding the status of maternal and child health care in Suizhou City during such period can provide the local health administrative department valid scientific bases upon which to construct effective policies.

**Methods:**

Various types of annual reports on maternal and child health care were collected and analyzed retrospectively.

**Results:**

Mortality rates for infants and children under 5 years showed a declining trend, while the rates of newborn home visiting, maternal health service coverage, and children health systematic management increased annually in Suizhou City from 2005 to 2011. The incidence of birth defect increased from 2.42‰ in 2005 to 3.89‰ in 2011. The maternal mortality ratio (MMR) fluctuated from 8.39/100,000 to 28.77/100,000, which was much lower than the national MMR (30.0/100,000 in 2010). The rates of hospitalized delivery and births attended by trained health personnel for pregnant women increased to more than 90% in the past five years.

**Conclusions:**

The improvements in maternal and child health care work in Suizhou City are worthy of recognition. Thus, the government should continue to increase funding in these areas to promote the complete enhancement of the maternal and child health care system.

## Introduction

Improving the health and well-being of women and children has remained a common goal throughout the world [1–3]. In 2001, the State Council of the People’s Republic of China developed and implemented “*Guidelines on Women’s Development in China* (*2001-2010*)” and “Guideline on Children’s Development in China (2001-2010)” [4–6]. These not only defined new targets and tasks for the development of health care systems for Chinese women and children, they also demonstrated that the government attaches great importance to the welfare of women and children. The main objectives of “Guideline on Women’s Development in China (2001-2010)” in the health section include the following: to ensure that women have access to health care services throughout their lifetime, improve the status of their reproductive health, guarantee their right to practice family planning, control the HIV infection rate to a relatively low level, and to raise the awareness of women regarding fitness and health [6]. The main objectives of “Guideline on Children’s Development in China (2001-2010)” in the health section include the following: to improve the quality of births, reduce the incidence of birth defects, improve the safety of maternal delivery, reduce the infant and under-five mortalities, improve childhood nutrition, and to strengthen the education of child health care [5]. The Suizhou Health Bureau has strengthened the management of the health care systems of women and children in order to achieve the abovementioned goals in the designated time [7].

**Figure 1:**
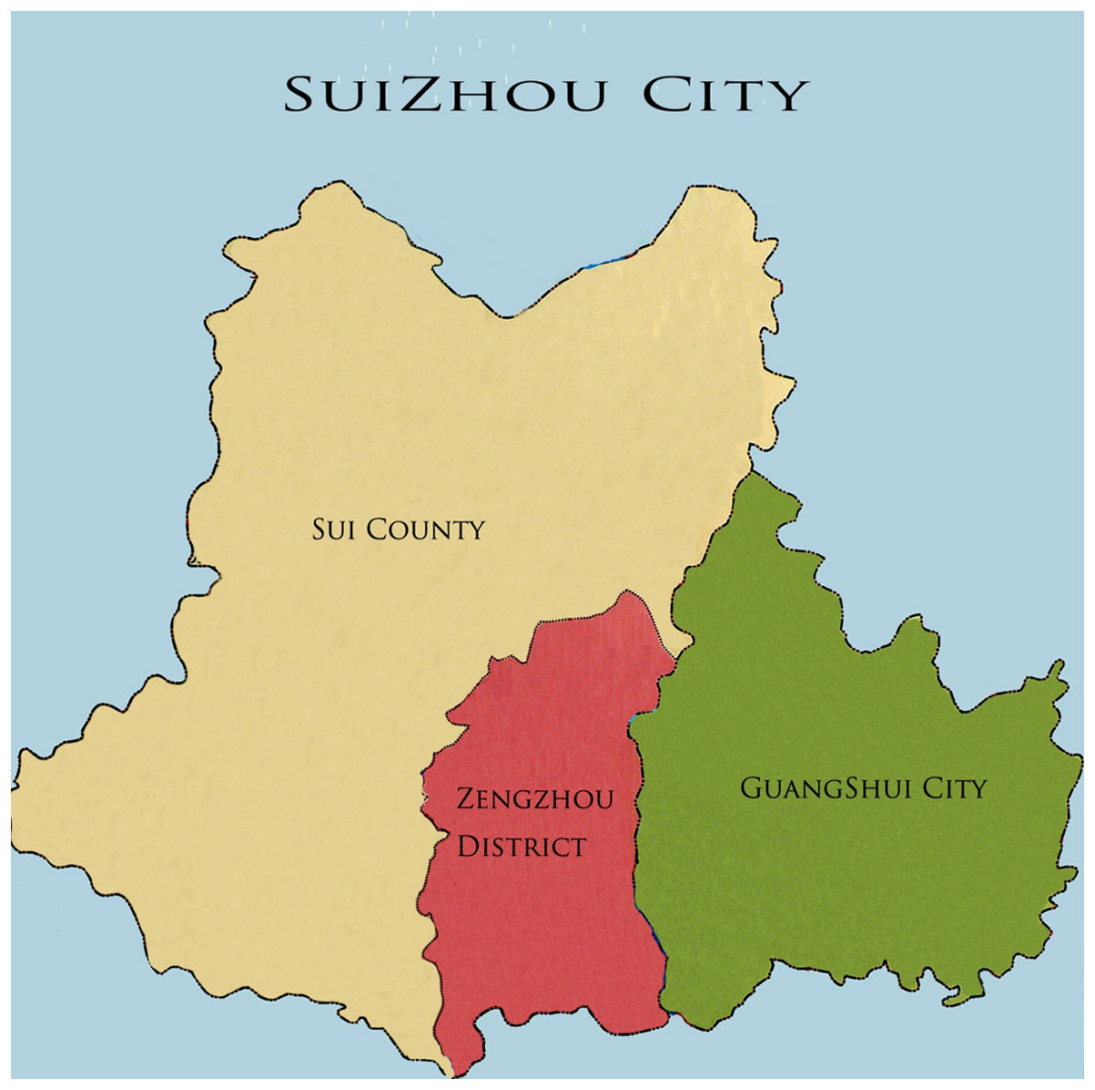
Suizhou City Map.

According to the *2010 Hubei Statistical Yearbook*, Suizhou City occupies a land area of 9,636 square kilometers and has a population of 2,577,700 people, of which 2,162,222 are considered residents. Suizhou City has jurisdiction over one district (*Zengdu District*), one county (*Sui County*), and one county-level city (*Guangshui City*) (Figure 1, Suizhou City Map). From 2005 to 2011, the average annual number of pregnant and parturient women was 22,405; that of infants was 22,477 and that of children under 5 years was 98,811. Reliable, complete, and timely information on maternal and child health (MCH) are essential for public health decision-making and action, including policy making, planning, programming, monitoring, and achievement of health-related goals [3]. In the present study, the MCH status from 2005 to 2011 in Suizhou City was analyzed to provide scientific bases with which to improve the health care system for women and children.

## Methods

### Data collection

Suizhou City has 66 medical health care organizations, including 50 health clinics in towns and 16 in urban areas. The departments devoted to women health care, child health care, and gynecology and obstetrics are responsible for the related works of MCH. All the data were encoded into a network system called “*National General Information Platform on Women and Children Health*” [8], which was established in China in 2005. This network system requires the MCH department in local cities or counties to install the *Direct Network Reporting Platform for MCH*, and to report and update the data every month.

The collection of information and data related to the status of MCH in Suizhou City from 2005 to 2011 was carried out through the hierarchical network of MCH services [9], and covered all pregnant and parturient women and children under 7 years in Suizhou City. MCH detailed information and data were first collected and reported by MCH personnel deployed in village clinics and health clinics located in towns. Data were subsequently reported to MCH facilities or hospitals at the county, district, and city levels. Finally, all the information and data were collected and reported to the *Suizhou Maternal and Child Health-care Hospital*. These included MCH annual report forms, maternal and child health care manuals, newborn home visiting manuals, report cards on maternal mortality, report cards on neonatal and puerperium death, report cards on deaths of children under 5 years, and report cards on recorded birth defects.

### Data quality control

The “National Maternal and Child Health Survey System” [10], “Monitoring and Assessment Guide for Development Platform of Chinese Women and Children,” and “*Hubei Province Maternal and Child Health Information Quality Control Guide*” were used as guides to monitor the reported data. All the recorders attended one-month courses before starting their work; they also attended bi-annual two-week refresher courses, and were qualified to fill in the report cards according to the National Health Statistics Work Management Approach [11].

**Figure 2:**
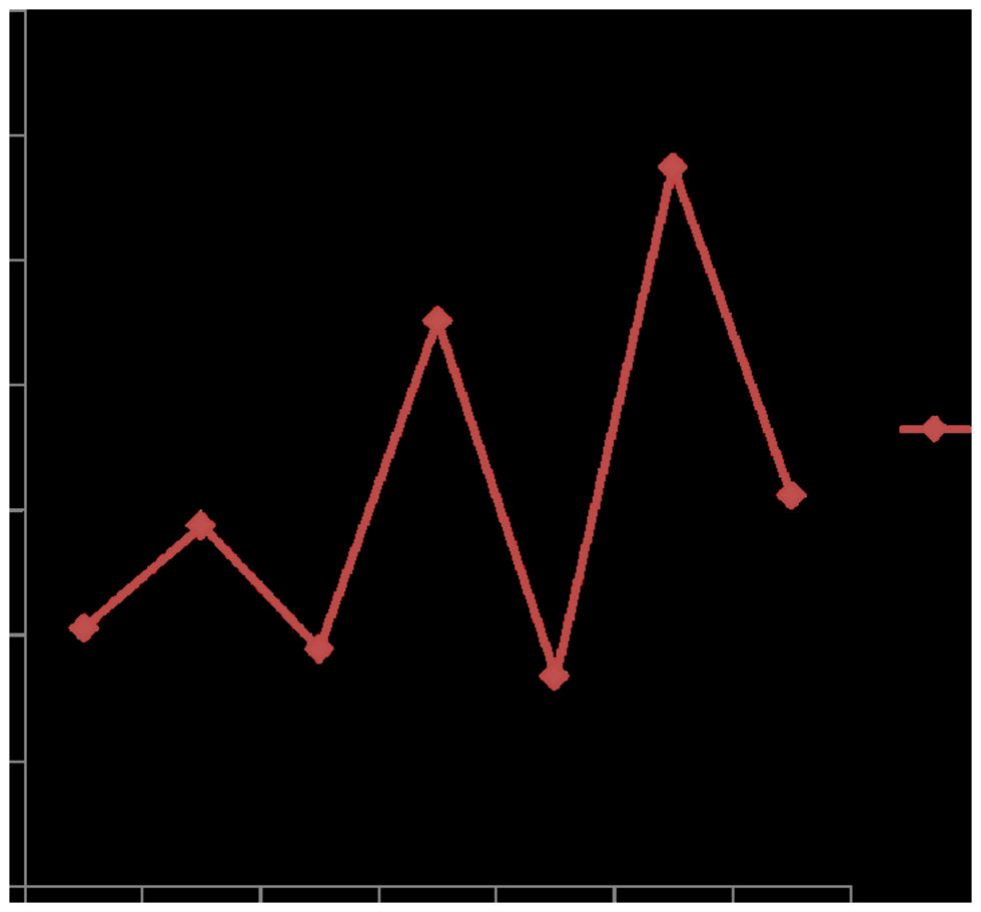
Maternal mortality ratio (deaths/100,000 live births) (2005 to 2011). Maternal mortality ratio (MMR) is the ratio of the number of maternal deaths per 100,000 live births from any causes related to or aggravated by pregnancy or its management, excluding accidental or incidental causes. Maternal death is the death of a woman during pregnancy or within 42 days after termination of pregnancy, irrespective of the duration and site of the pregnancy, from any causes related to or aggravated by the pregnancy or its management, but not from accidental or incidental causes.

In addition, the *Suizhou Maternal and Child Health-care Hospital* also holds regular meetings together with community health service facilities, hospitals in towns, and village clinics every season, with the purpose of inspecting the data quality. The City Health Bureau likewise organizes spot checks twice every year.

### Ethical review

The study protocol was approved by the institutional review boards of the Tongji Medical College, Huazhong University of Science and Technology. All the participants signed the informed written consent after the MCH personnel explained the study protocol to them.

## Results

### Maternal health service indicators

From 2005 to 2011, maternal health service coverage, systematic maternal health care management, rate of births attended by trained personnel, and rate of hospitalized deliveries were maintained at a high level in Suizhou City. With the enhancement of the management system, the systematic maternal health care management has recorded an improvement rate of 85% since 2008. In addition, Maternal HIV screening rate increased from 65.30% in 2005 to 98.86% in 2011 (Table 1).

**Table 1:** Utilization of Maternal Health Care Services.

Year	Total Number of Pregnancy	Maternal Health Service Coverage		Systematic Maternal Health Care Management		Births Attended by Trained Health Personnel		Hospitalized Delivery		Maternal HIV Detection	
		Number	Rate (%)	Number	Rate (%)	Number	Rate (%)	Number	Rate (%)	HIV(+) Number	Detection Rate (%)
2005	19302	18124	93.90	15658	81.12	19182	99.38	18889	97.86	2	65.30
2006	20799	19247	92.54	15649	75.24	20580	98.95	20362	97.90	1	86.12
2007	21136	20402	96.53	16488	78.01	21136	100.00	20960	99.17	3	90.74
2008	22008	20683	93.98	18836	85.59	21994	99.94	21971	99.83	1	90.80
2009	23779	22599	95.04	21389	89.95	23779	100.00	23772	99.97	5	91.20
2010	24301	23530	96.83	22267	91.63	24296	99.98	24294	99.97	6	92.41
2011	25513	24643	96.59	22785	89.31	25500	99.95	25500	99.95	4	98.86

**Maternal health service coverage rate (%)**: (The number of women who took part in maternal health service in a year / the total number of pregnant and parturient women during the year) × 100%.

**Systematic maternal health care management rate (%)**: (The number of women who took part in systematic maternal health care management in a year / the total number of pregnant and parturient women during the year) × 100%.

**maternal health care management** refers to the number of pregnant women who receive early pregnancy check-ups, prenatal examinations (not less than seven times in the city, not less than four times in rural area), births attended by trained health personnel, and postpartum visit (at least once) during pregnancy and within 28 days postpartum.

**Rate of births attended by trained health personnel (%)**: (The number of births attended by trained primary medical staffs or midwives in a year / the total number of live births during a year) × 100%.

### Maternal death status

From 2005 to 2011, maternal mortality ratio (MMR) fluctuated from 8.39/100,000 to 28.77/100,000 (Figure 2), remaining at a low level compared with the national MMR of 30.0/100,000 in 2010 [12]. In 2011, four maternal deaths were recorded. These were caused by septic shock with severe pancreatitis after cesarean section, pregnancy associated with acute lymphoblastic leukemia, hemorrhagic shock with respiratory failure after cesarean section, and preeclampsia with placental abruption and intraventricular hemorrhage.

### Child health service indicators

From 2005 to 2011, all newborn home visiting rates were above 90%. With the strengthened management of the children health system, the health systematic management rate for children under 3 years increased from 74.30% in 2005 to 93.13% in 2011. Furthermore, the coverage rate for children under 7 years increased from 73.71% in 2005 to 92.75% in 2011. On December 6, 2007, Suizhou City officially launched the newborn disease screening program, with the goal of screening for phenylketonuria, hypothyroidism, and hearing impairment. Since its implementation, the newborn disease screening rate increased from 19.72% in 2008 to 62.24% in 2011 (Table 2).

**Table 2:** Utilization of Child Health Care Services.

Year	Newborn Home Visiting		Newborn Disease Screening		Children Under-3 Health Systematic Management		Children Under-7 Health Service Coverage	
	Number	Rate (%)	Number	Rate (%)	Number	Rate (%)	Number	Rate (%)
2005	18284	94.23	0	0.00	41867	74.30	84946	73.71
2006	19595	93.88	0	0.00	42234	71.02	82410	71.08
2007	19634	92.59	0	0.00	40538	64.04	85752	68.86
2008	21353	96.62	4358	19.72	57201	80.70	108684	79.78
2009	22964	96.39	10118	42.47	62587	89.16	125487	91.05
2010	23575	96.88	12386	50.90	69244	91.60	135960	93.93
2011	24779	96.81	15931	62.24	71932	93.13	138828	92.75

**Newborn home visiting rate (%)**: (The number of newborns who receive newborn home visits at least once in a year / the total number of live births during the year) × 100%.

**The rate of children under-3 health systematic management (%)**: (The number of children under-3 who took part in child health systematic management in a year / the total number of children under-3 during the year) × 100%.

**Children under-3 health systematic management** refers to the health check-ups for children under-three, including four times for children under 1-year-old (3, 6, 9, and 12 months) and twice per year for 1- to 3-year-old children (18, 24, 30, and 36 months).

**The rate of children under-7 health service coverage (%)**: (The number of children under-7 who took part in child health service at least once in a year / the total number of children under-7 during the year) × 100%.

Table 3. Screening for Common Gynecological Diseases

### Child death status

**Figure 3:**
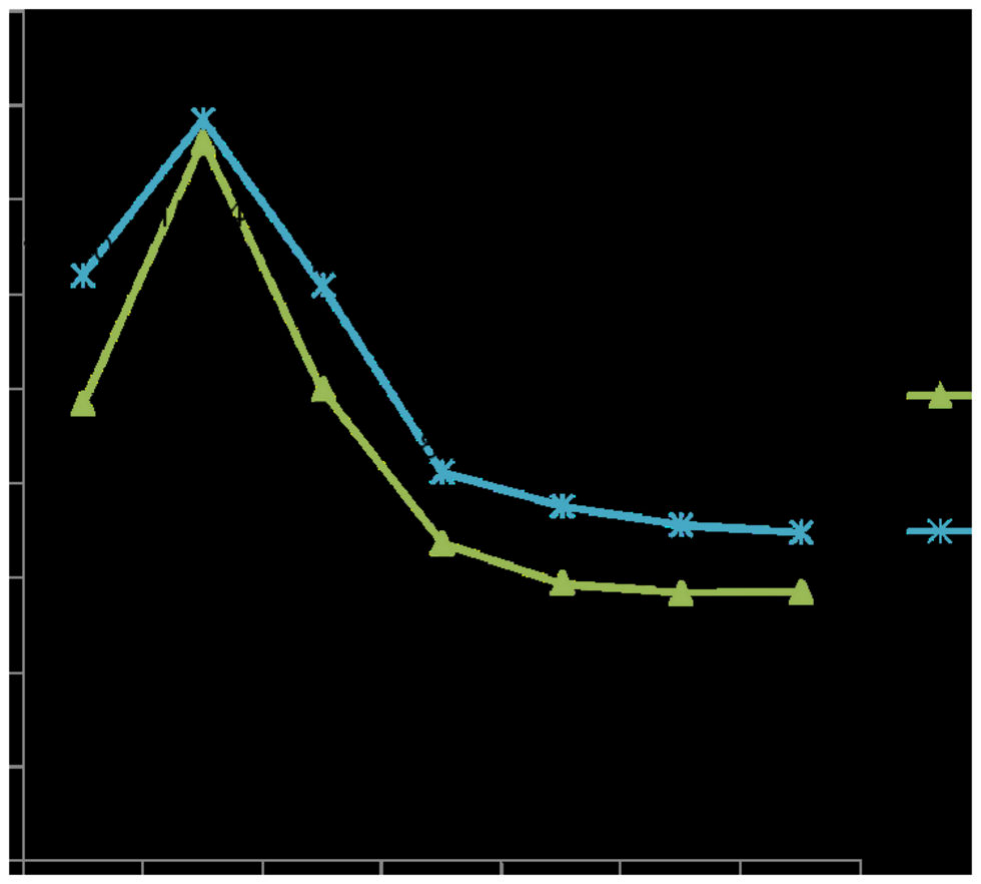
Infant mortality rate (deaths/1,000 live births) and children under-five mortality rate (deaths/1,000 live births) (2005 to 2011). **Infant mortality rate (IMR)** is the number of deaths of babies under one year of age per 1,000 live births per year. **Children under-five mortality rate (U5MR)**: the number of children who die by the age of five, per 1,000 live births per year.

**Figure 4:**
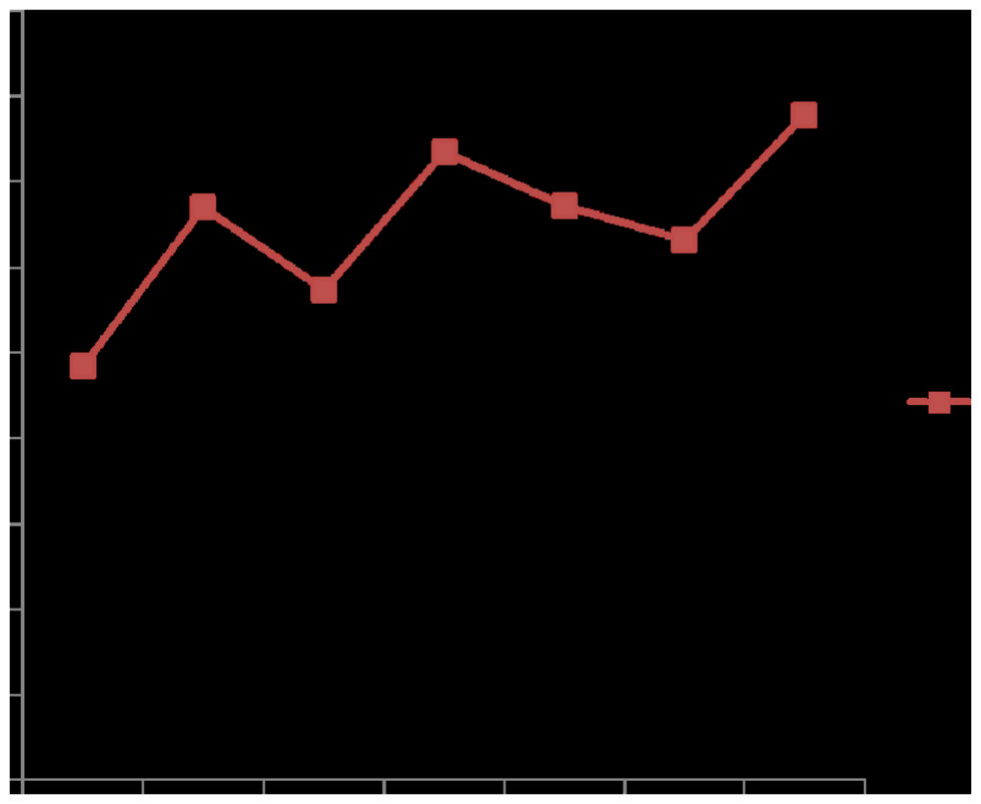
Birth defect incidence(‰) (2005 to 2011). Birth defect incidence(‰): (The number of birth defects in a year / the total number of perinatal including live births, death-births and stillbirths) × 1000‰.

Infant mortality rate decreased from 15.24/(1,000 live births) in 2006 to 5.70/(1,000 live births) in 2011. The mortality rate for children under 5 years also decreased from 15.70/(1,000 live births) in 2006 to 6.95/(1,000 live births) (Figure 3). The leading causes of deaths among children under 5 years included congenital heart disease, preterm/low birth weight births, accidental suffocation, neonatal asphyxia, pneumonia, and drowning. The incidences of birth defects increased, rising from 2.42‰ in 2005 to 3.89‰ in 2011 (Figure 4). The top five malformations in 2011 included cleft lip and palate, deformities in the fingers and foot, congenital heart disease, congenital hydrocephalus, and external ear malformation.

### Common gynecological diseases screening status

The screening subjects of common gynecological diseases included female residents aged 20 years to 64 years. The diseases that were screened included vaginitis, cervicitis, condyloma acuminatum, cervical carcinoma, breast cancer, and oophoroma [10]. From 2005 to 2011, the screening rates of common gynecological diseases fluctuated from 15.16% to 40.87%, and the illness incidence rates in the subjects fluctuated from 21.77% to 41.36% (Table 3).

**Table 3:** Screening for Common Gynecological Diseases


Year	2005	2006	2007	2008	2009	2010	2011
Screening Rate (%)	24.77	22.39	19.85	15.16	36.12	40.87	19.61
Illness Incidence Rate (%)	21.77	23.5	33.51	22.51	30.3	41.36	34.72
Incidence of Trichomonas Vaginitis (%)	7.38	7.5	8.29	11.8	13.23	21.4	17.3
Incidence of Cervical Erosion (%)	11.69	11.75	14.24	9.86	13.61	17.18	12.07

**Common gynecological diseases screening rate (%):** (The number of women who took part in women common diseases screening in a year / the total number of women that should have taken part in women common diseases screening during the year) × 100%.

## Discussion

The health and well-being of mothers, infants, and children are of critical importance; these are reflections of the current health status of individuals, local communities, and the nation as a whole, as well as predictors of the health of the next generations to come [13]. In recent years, an increasing number of women and children have enjoyed quality MCH services in China, because of the gradually expanded and enlarged scope of MCH services [12]. The Chinese government established the annual report system of information pertaining to women and children’s health in the early 1980s, which was followed by the “National General Information Platform on Women and Children Health” in 2005 [14]. With the development of electronic information technology, the means of monitoring and reporting medical data have greatly improved. All information on women and children’s health can be reported directly online. In addition, the quality and efficiency of reported information has also greatly improved.

Suizhou City has long recognized the importance of maternal and child health care, and has made tremendous efforts to improve the quality of such care. For example, in 2001, the Suizhou government held several thematic conferences and set up a leading group for the MCH work of the two guidelines in 2001. Likewise, the Municipal Health Bureau and subordinate units set up leading groups to assist and support the work of the Women and Children Working Committee. Since 2001, MCH institutions have carried out the “*National Health Education Activities*,” and publicized the prevention measures of the “*Improve the Quality of Births, Reduce Birth Defects and Disabilities*” program by posting in bulletin board postings; raising information awareness through radio, television, and newspapers; and establishing free clinics. In addition, the “*Build MCH Advanced Villages and Towns*” and “*Maternal-Neonatal Health and Safety*” activities were also conducted, which strengthened the development of community health service facilities and set up a leading group to prevent mother-to-child transmission of AIDS since 2005.

Thus far, a complete set of health care services to systematically monitor maternal health includes prenatal care, prenatal screening and diagnosis, screening and management of high-risk pregnant women, hospitalized delivery, newborn care, and postpartum visits. From 2005 to 2011, the utilization of maternal health service was fairly high in Suizhou City, with the coverage rate reaching more than 90% since 2005. Nevertheless, the systematic maternal health care management can still be strengthened and improved further, with a systematic management rate of 89.31% in 2011 (lower than 91.63% in 2010, although higher than 84.1% of national rate in 2010) [12]. Since 2005, Suizhou City has also carried out the “*Reduce and Eliminate Maternal Mortality Project*,” especially by implementing an enhanced MCH project since 2009, which prioritized investigations into the main causes and influencing factors of maternal death. As a result, the MMR has been maintained at a low level in Suizhou City [7].

Meanwhile, since 2001, the Suizhou Health Bureau stressed the importance of health care management for preschool children and systematic management for children, and integrated management of childhood illness, in accordance with the “Guideline on Children’s Development in China (2001-2010).” The utilization of child health services has gradually increased. Since 2009, the rate of health service coverage for children under 7 years has increased to over 90%, which is higher than the 83.4% national rate in 2010. However, the Health Bureau still needs to improve its service and strive to cover more children in Suizhou.

In China, normal full-term newborns receive at least two home visits by MCH personnel. The first visit is conducted 7 days after discharge from hospital, whereas the second visit is usually done 28 days to 30 days after birth (i.e., full-moon visit). MCH personnel increase the frequency of visits for the high-risk newborns depending on the condition of the infant, and conduct their first visit within 3 days after discharge. Home visits mainly include inquiry, physical examination, and provision of guidance to the parents regarding newborn care. Newborn home visiting rates have increased by up to 96% since 2008; however, the Health Bureau must still broaden the newborn home visiting coverage, given that a small percentage of newborns are still being delivered at home. Broadening such coverage can also increase newborn disease screening. The development of perinatology and preventive medicine, the improvement of the pediatric and neonatal transfer system, the emphasis of the government and relevant departments on MCH, and the improvement of parents’ awareness of health care have all resulted in the declining trend of both infant and under-five mortality.

In 2011, birth defect incidence was recorded at 3.89‰, lower than the national incidence of 15.325‰. The decreasing incidence of birth defects can be attributed to environmental factors, the improvement of MCH network, and the improved quality of premarital health care work (including premarital health guidance, counseling, and medical check-up services provided for men and women who plan to get married) [12]. The provision of medical advice and guidance by doctors is also important in reducing birth defects because it raises the health awareness of pregnant women. For example, the Health Bureau must convince more women to take folic acid to prevent neural tube defects and avoid environmental risk factors before pregnancy and during early pregnancy. In addition, hospitals should also continue to improve the technical merit of prenatal diagnosis [15].

In 2006, the Suizhou government incorporated the Preventing Mother-to-Child Transmission (PMTCT) of AIDS, syphilis, and hepatitis B in major maternal and child health care projects. The detection rates steadily increased as PMTCT of AIDS was brought into the routine work of MCH management, along with the number of HIV-positive women. From 2005 to 2010, a total of 18 HIV-positive pregnant women were identified to be young women at the age of 21 years to 29 years who contracted HIV through sexual transmission in Suizhou City. Efforts to prevent the mother-to-child transmission of AIDS still face severe difficulties and challenges.

The rate of diagnosis of common gynecological diseases, as well as the actual number of affected women, has steadily increased over the years. The screening rate of common gynecological diseases was only 19.61% in 2011, lower than the rate of 40.87% in 2010. However, the actual incidence in women might be higher than the data shown. Given that common gynecological diseases are a critical factor affecting the safety and life quality of women, the Health Bureau and relevant departments must prioritize the general survey of women’s health. The Bureau should also consider the screening and treatment of gynecological diseases as one of the major aspects of health care work directed towards women, so that these women can receive high-quality medical services and benefit from the early prevention of disease [12].

MCH staff regularly visited all the hospitals in Suizhou City to inspect the medical records every month; hence, data on maternal deaths, child deaths, and live births were not under-reported. However, the number of birth defects might be under-reported, given that data were obtained only from report cards on birth defects as reported by MCH personnel in towns. In addition, some cases of birth defects not directly diagnosed following birth (e.g., congenital heart disease) were not reported. For example, birth defects in Suizhou City are only reported from the department of gynecology and obstetrics; however, birth defects that might be discovered in pediatric or neonatology departments are not reported.

The present study has several limitations. The surveillance population in the study consisted of all the pregnant and parturient women as well as preschool children in Suizhou City. Therefore, confidence intervals or other measures of uncertainty were not established. In addition, data from urban and rural areas were not separated during data collection. Therefore, a comparison of the quality of MCH services between urban and rural areas was not conducted. Likewise, the relationship between MCH mortality and service utilization and quality was not investigated, because it was assumed that MCH was unlikely due to the small number of deaths. The monitored data of the MCH system may be lower than the actual data because the data collection activities of the family planning department and health department are mutually independent in China. For example, women usually consult the family planning department in order to screen for common gynecological diseases instead of the MCH department. This can lead to reduced quality and data integrity of MCH services. In the future, the family planning department and MCH department should fully coordinate with each other in implementing cross-service and information exchange.

Based on ample evidence, the widespread improvement of maternal and child health care system continues to be of critical importance. Suizhou City still has a long way to achieve the ideal maternal and child health and well-being conditions. The local government must be ready to adjust the management mode and catalyze new methods around the best evidence and practices for maternal and child health care [3,13,16,17]. Furthermore, the local government should assess the strengths, weaknesses, and possible barriers of improvement in the current system; identify targets for policy-making; and develop a blueprint for action that would lead to widespread and sustainable system-wide improvements in maternal and child health care programs and outcomes [1–3,16,18].

## Conclusions

The utilization of MCH services in Suizhou City has steadily increased over the years. With the enhancement and improvement of systematic health management, the MMR, child mortality rate, and birth defect incidences were maintained at a low level from 2005 to 2011. Nevertheless, the improvements in maternal and child health care services in Suizhou City are worthy of recognition. The Suizhou Government and Health Bureau should continue to increase funds allocated for promoting the complete enhancement of MCH.
